# Comparative dataset on productivity, proximate, biochemical and pigment content of marine *Gonyostomum* sp. and freshwater *Tetraedron* sp. microalgae

**DOI:** 10.1016/j.dib.2024.110393

**Published:** 2024-04-05

**Authors:** Proma Dey, Jannatul Nayeem, Sumit Kanti Dey, Dipa Debi, Nusrat Zaman Zemi, S. M. Rashadul Islam, Rezaul Karim, Helena Khatoon

**Affiliations:** a Department of Aquaculture, Chattogram Veterinary and Animal Sciences University, Chattogram 4225, Bangladesh; b Institute of Technology Transfer and Innovation, Bangladesh Council of Scientific and Industrial Research, Dr Qudrate-I-Khuda Road, Dhaka-1205, Bangladesh

**Keywords:** *Gonyostomum* sp., *Tetraedron* sp., Productivity, Proximate composition, Biochemical composition, Pigment

## Abstract

The dataset includes a comparative analysis of *Gonyostomum* sp. and *Tetraedron* sp. to characterize their productivity, proximate composition, biochemical composition and pigments. Growth data were collected through cell density and optical density and subsequently mass-cultured to utilize biomass for other analyses. The onset of the stationary phase (12 to 18 days) varied between the species. Volumetric productivity, areal productivity, and SGR were also significantly higher (*p* ˂ 0.05) in *Gonyostomum* sp. whereas, *Tetraedron* sp. showed significantly higher (*p* ˂ 0.05) cell duplication time and cell doublings per day (K). *Gonyostomum* sp. showed significantly higher (*p* ˂ 0.05) protein (42.86±1.13%), carbohydrate (13.56±0.48%) and lipid (27.4 ± 0.69%) content than *Tetraedron* sp. Significantly higher (*p* ˂ 0.05) polyunsaturated fatty acids (PUFA) were obtained from both *Gonyostomum* sp. and *Tetraedron* sp. Non-essential amino acids were prevalent in both microalgae than essential amino acids. Significantly higher (*p* ˂ 0.05) chlorophyll-a (5.51±0.00), chlorophyll-b (2.27±0.04) and phycobiliprotein (2.32±0.05) were found in *Tetraedron* sp. Conversely, *Gonyostomum* sp. exhibited higher (*p* ˂ 0.05) carotenoid content (2.48±0.05). These findings may contribute to the screening and utilization of these microalgae in the aquaculture, pharmaceuticals, and nutraceuticals sectors.

Specifications Table

**Table:** 

Subject	Food Science, Aquatic Science
Specific subject area	Comparative data on productivity, proximate, biochemical and pigment content of marine (*Gonyostomum* sp.) and freshwater (*Tetraedron* sp.) microalgae
Data format	Raw and analyzed primary data
Type of data	Picture, Graph and Table
Data collection	Cell density and optical density data were assessed spectrophotometrically to determine the growth phases. Data on growth and productivity parameters were calculated through cell count, biomass, culture volume and lipid data Proximate composition was assessed using chemical methods. For fatty acids: Gas Chromatography Mass Spectrophotometry GCMS analysis For amino acids: SYKAM amino acid analysis of *Oscillatoria* spp.; essential amino acids, non-essential amino acids. For chlorophyll-a content: spectrophotometric analysis at 750 nm, 664 nm, 647 nm, and 630 nm wavelengths. For carotenoid: spectrophotometric analysis at 450 nm. For phycobiliprotein: Phycocyanin, allophycocyanin, phycoerythrin (562 nm, 615 nm, 652 nm, and 720 nm) The acquired data were further analyzed through MS Excel and IBM SPSS (v. 26.0) software.
Data source location	Microalgae Research Corner; Disease and Microbiology Laboratory, Department of Aquaculture, Faculty of Fisheries, Chattogram Veterinary and Animal Sciences University (CVASU), Khulshi-4225, Chattogram, Bangladesh
Data accessibility	Data are available with this article and also at Repository name: Mendeley Data Data identification number: Direct URL to data: https://data.mendeley.com/datasets/pwkgyshw4s/1

## 1 Value of the Data

• Growth and productivity data along with proximate composition and amino acid data will be helpful to incorporate the potential microalgae into aquafeed as partial or complete substitutes.
• Fatty acid data is crucial to explore the microalgal potency in fish nutrition and biofuel production. Pigment content also serves the purpose of enhancing coloration in valuable commercial and ornamental fish.
• Comparative analysis between freshwater and marine microalgae serves as the groundwork for screening potential microalgae and their specific utilization in aquaculture, pharmaceuticals, and nutraceuticals industries.

## 2 Background

Microalgae have recently been attracted considerable interest worldwide, due to their extensive application potential in the renewable energy, biopharmaceutical, and nutraceutical industries [2]. Microalgae have the potentiality to reduce reliance on conventional raw materials. Their high nutritional quality, positive effects on growth, increased muscle triglycerides and proteins, disease resistance, reduced nitrogen output, and omega-3 fatty acids contribute to improved carcass quality in aquatic species [3]. Microalgae also present excellent advantages, like high growth rates, high productivity, no requirement of agricultural land for their cultivation, short harvest cycles, ease of cultivation, high lipid content, and high photosynthetic efficiency [4]. To utilize microalgae commercially in aquaculture, pharmaceuticals and other sectors, screening of microalgal species should be done through performance characterization. However, very little research has been conducted on the characterization of microalgae in Bangladesh. Therefore, the present approach is to compare the growth, productivity, nutritional, biochemical and pigment properties of marine (*Gonyostomum* sp.) and freshwater (*Tetraedron* sp.) microalgae which may contribute to select potential strains that will offer competitive advantages for any commercial use.

## 3 Data Description

**Fig. 1:**
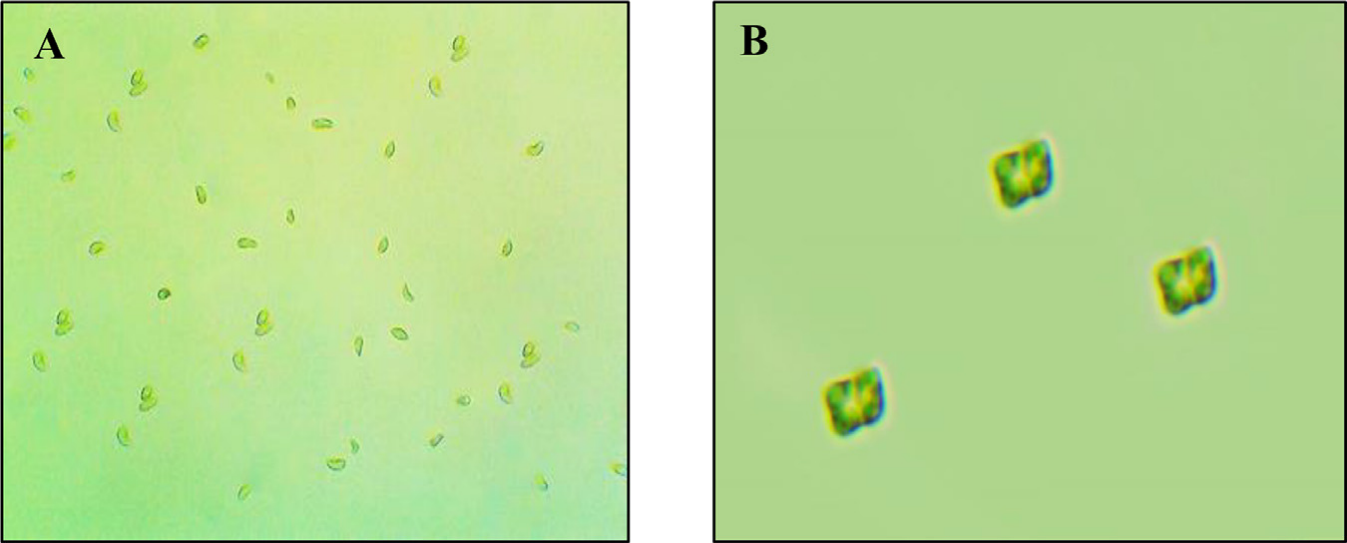
Microscopic view of microalgae, *Gonyostomum* sp (A). and *Tetraedron* sp. (B).

Comparative analysis of two microalgae including *Gonyostomum* sp., *Tetraedron* sp. (Fig. 1) is presented in this dataset along with their growth and productivity parameters; nutritional composition; biochemical (fatty acid and amino acid) profile and pigment (chlorophyll, carotenoid and phycobiliprotein) content [1].

**Fig. 2:**
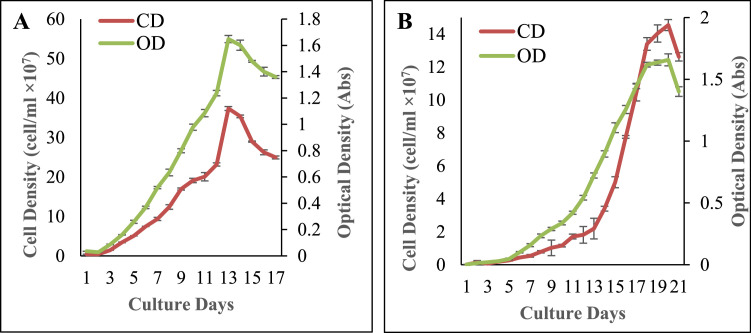
Growth curve in terms of cell density (cells/ml × 10^7^) and optical density (Absorbance) of marine and freshwater microalgae, *Gonyostomum* sp. (A), *Tetraedron* sp. (B). Values are mean ± standard error. CD and OD represent cell density and optical density, respectively.

Growth phases of *Gonyostomum* sp., *Tetraedron* sp. were determined in terms of cell density (cells/ml × 10^7^) and optical density (Absorbance). Fig. 2 illustrates the cell density and optical density of each species as a function of culture time. *Gonyostomum* sp. showed lag phase and exponential phase on day 1–2 and 2 to 12, respectively. It also showed the stationary phase on days 12 to 13, and the death phase commenced from day-13. *Tetraedron* sp. exhibited lag phase, exponential phase and stationary phase on day 1 to 4, day 4 to 18 and day 18 to 20 was recorded as, respectively. *Gonyostomum* sp. displayed significantly higher [t (4) =14.466, *p* = 0.00] cell density (23.17±0.41 cells/ml × 10^7^) and OD value (1.65±0.03) on 12th day compared to *Tetraedron* sp.

Growth and productivity parameters varied between *Gonyostomum* sp. and *Tetraedron* sp. (Table 1).) *Gonyostomum* sp. showed significantly higher SGR [t (4) = 2435.253, *p* = 0.00], cell density [t(4) =14.466, *p* = 0.00], volumetric productivity [t (4) = 499.992, *p* = 0.00], areal productivity [t(4) = 499.992, *p* = 0.00] and lipid productivity [t (4) = 20.036, *p* = 0.00] than *Tetraedron* sp. Contrarily, *Tetraedron* sp. resulted in significantly higher cell duplication time [t (4) = −2446.272, *p* = 0.00] and cell doublings per day (K) [t (4) = −2.889, *p* = 0.04] than *Gonyostomum* sp.

**Table 1:** Growth and productivity analysis of *Gonyostomum* sp. and *Tetraedron* sp.

Growth and Productivity Factors	*Gonyostomum* sp.	*Tetraedron* sp.
Cell duplication time (Day)	1.12±0.00^b^	1.86±0.00^a^
SGR (mg/day)	0.62±0.00^a^	0.37±0.00^b^
Cell doublings per day (K)	0.48±0.00^b^	0.52±0.01^a^
Cell Density (cells/ml × 10^7^)	23.17±0.41^a^	13.53±0.52^b^
Volumetric productivity (mg/L/Day)	30.24±0.03^a^	13.07±0.01^b^
Areal productivity (mg/cm^2^/day)	3.02±0.00^a^	1.31±0.00^b^
Lipid Productivity (mg/L/Day)	8.29±0.22^a^	3.34±0.12^b^

**Fig. 3:**
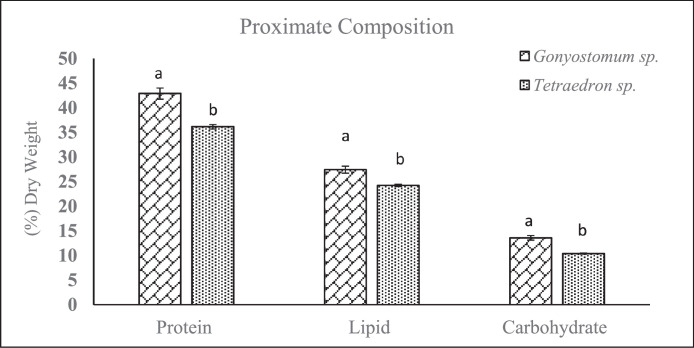
Proximate composition (% dry weight) including protein content, lipid content and carbohydrate content of *Gonyostomum* sp. and *Tetraedron* sp. Values are the mean of the triplicates with standard error bar (means ± SE).

The significant variations of the proximate composition of *Gonyostomum* sp. and *Tetraedron* sp. are depicted in Fig. 2 where the crude protein, lipid and carbohydrate contents are determined from the dried biomass. Significantly higher protein [t (4) = 5.553, *p* = 0.005] was obtained from *Gonyostomum* sp. (42.86±1.13%) than *Tetraedron* sp. (36.12±0.45%). *Gonyostomum* sp. also showed significantly higher carbohydrate [t (4) = 6.487, *p* = 0.003] and lipid [t (4) = 4.382, *p* = 0.012] than *Tetraedron* sp. which accounted for 13.56±0.48% and 27.4 ± 0.69%, respectively (Fig. 3).

Fatty acid profile (mean± SE) of *Gonyostomum* sp. and *Tetraedron* sp. is demonstrated in Table 2. Saturated fatty acids, monounsaturated fatty acids and polyunsaturated fatty acids were found significantly variable among the species. Ratios of fatty acid content were also statistically significant (*p* < 0.05) between *Gonyostomum* sp. and *Tetraedron* sp.

**Table 2:** Fatty acids content (% total) of *Gonyostomum* sp. and *Tetraedron* sp., expressed as the average of the duplicates with standard error bar (mean± SE).

Carbon	Fatty Acid Methyl Esters	*Gonyostomum* sp.	*Tetraedron* sp.
		Conc (ppm)	
C8:0	Methyl Octanoate	1.63±0.02	1.17±0.00
C10:0	Methyl Decanoate	1.47±0.02	0.93±0.05
C12:0	Methyl Laurate	5.05±0.02	4.64±0.00
C13:0	Methyl Tridecanoate	0.40±0.00	0.54±0.00
C14:0	Methyl Myristate	0.17±0.02	0.06±0.00
C16:0	Methyl Palmitate	2.22±0.03	5.47±0.32
C18:0	Methyl Stearate	0.57±0.26	0.45±0.38
C20:0	Methyl Arachidate	8.23±0.46	11.97±0.92
C17:0	Methyl Heptadecanoate	4.24±0.02	3.74±0.00
C21:0	Methyl Heneicosanoate	0.02±0.01	0.02±0.01
C22:0	Methyl Behenate	0.00±0.00	0.00±0.00
C23:0	Methyl Tricosanoate	0.18±0.11	0.00±0.00
C24:0	Methyl Lignocerate	0.00±0.00	0.00±0.00
	**ƩSAFA**	**24.17±0.72^b^**	**28.98±0.94^a^**
C16:1	Methyl Palmitoleate	20.19±0.04	28.71±0.73
C18:1	Methyl Oleate	2.31±0.00	1.16±0.15
C20:1	Methyl cis-11-eicosenoate	3.01±0.00	0.00±0.00
C22:1	Methyl Erucate	3.09±0.02	2.07±0.01
C24:1	Methyl Nervonate	0.00±0.00	0.01±0.01
	**ƩMUFA**	**28.60±0.03^b^**	**31.95±0.89 ^a^**
C18:2n-6	Methyl Linoleate	22.17±0.07	22.24±0.55
C20:3n-6	Methyl 11-14-17- Eicosatrienoate	0.24±0.03	0.55±0.01
C20:4n-6	Methyl Arachidonate	3.28±0.09	0.87±0.02
	**Ʃn6-PUFA**	**25.69±0.01^a^**	**23.66±0.54^b^**
C18:3n-3	Methyl Linolenate	11.22±0.85	8.17±0.25
C20:5n-3	Methyl icosa-5,8,11,14,17-pentaenoate	7.60±0.03	4.13±0.04
C22:5n-3	Methyl Docosapentaenoate	2.62±0.38	2.19±0.01
C22:6n-3	Methyl Docosahexanoate	0.10±0.00	0.11±0.00
	**Ʃn3-PUFA**	**21.54±1.21^a^**	**14.61±0.19^b^**
	**ƩPUFA**	**47.23±1.20^a^**	**38.27±0.35^b^**
	**Ʃn3/ Ʃn6**	**0.84±0.05^a^**	**0.62±0.02^b^**
	**DHA/EPA**	**0.01±0.00^b^**	**0.03±0.00^a^**
	**SAFA/TUFA**	**0.32±0.01^b^**	**0.41±0.02^a^**
	**SAFA/TFA**	**0.24±0.01^b^**	**0.29±0.01^a^**
	**TUFA/TFA**	**0.76±0.01^a^**	**0.71±0.01^b^**

Amino acid contents (% amino acid) of *Gonyostomum* sp. and *Tetraedron* sp. are presented in Table 3. Non-essential amino acids [t (4) = 5.801, *p* = 0.028] were found more prevalent than essential amino acids [t (4) = −6.233, *p* = 0.025] in both *Gonyostomum* sp. and *Tetraedron* sp.

**Table 3:** Amino acid content (% amino acids) of *Gonyostomum* sp. and *Tetraedron* sp. (mean±SE). Here, **EAA**= Essential Amino Acid, **NEAA**= Non-Essential Amino Acid, **AA**= Total Amino Acid.

Compound Name (570 nm)	Types	*Gonyostomum* sp.	*Tetraedron* sp.
		Amount (%)	
Histidine	EAA	4.08±0.04	4.45±0.06
Isoleucine	EAA	2.21±0.05	2.21±0.01
Leucine	EAA	8.36±0.08	8.35±0.05
Lysine	EAA	5.71±0.09	6.19±0.04
Methionine	EAA	1.66±0.04	1.54±0.05
Phenylalanine	EAA	4.15±0.03	4.11±0.07
Threonine	EAA	5.91±0.06	5.63±0.09
Tyrosine	EAA	3.52±0.05	3.88±0.05
Valine	EAA	4.05±0.02	4.87±0.02
	ΣEAA	39.65±0.25^b^	41.23±0.07^a^
Alanine	NEAA	10.25±0.04	9.14±0.04
Arginine	NEAA	6.36±0.05	5.34±0.04
Aspartic acid	NEAA	12.28±0.02	12.05±0.05
Glutamic acid	NEAA	13.48±0.09	13.58±0.02
Glycine	NEAA	7.49±0.04	7.59±0.04
Cysteine	NEAA	0.03±0.02	0.48±0.04
Serine	NEAA	5.48±0.02	5.59±0.02
Proline	NEAA	4.98±0.02	4.99±0.02
	ΣNEAA	60.33±0.26^a^	58.76±0.06^b^
	ƩAA/ƩEAA	2.52±0.02 ^a^	2.43±0.00^b^
	ƩAA/ƩNEAA	1.66±0.01^b^	1.70±0.00^a^
	ƩEAA/ƩNEAA	0.66±0.01^b^	0.70±0.00^a^

Pigment contents exhibited significant variations between *Gonyostomum* sp. and *Tetraedron* sp. (Fig. 2). Chlorophyll-a [t (4) = −58.367, *p* = 0.00] and Chlorophyll-b [t (4) = −32.612, *p* = 0.00] contents were found significantly higher in *Tetraedron* sp. than *Gonyostomum* sp. Phycobiliprotein [t (4) = −8.394, *p* = 0.001] content was also found significantly higher in *Tetraedron* sp. than *Gonyostomum* sp. Conversely, Carotenoid [t (4) = 6.202, *p* = 0.003] content was found significantly higher in *Gonyostomum* sp. than *Tetraedron* sp. (Table 4).

**Table 4:** Pigment contents (means ± SE) of *Gonyostomum* sp. and *Tetraedron* sp.

Pigment Content	Gonyostomum sp.	Tetraedron sp.
Chlorophyll-a content (µg/ml)	3.19±0.04^b^	5.51±0.00^a^
Chlorophyll-b content (µg/ml)	0.88±0.02^b^	2.27±0.04^a^
Carotenoid content (µg/ml)	2.48±0.05^a^	2.06±0.04^b^
Phycocyanine content (mg/g)	0.33±0.04^b^	0.67±0.01^a^
Allophycocyanin content (mg/g)	1.05±0.05^a^	1.03±0.04^a^
Phycoerythrin content (mg/g)	0.41±0.03^b^	0.62±0.01^a^
Total phycobiliproteins content (mg/g)	1.79±0.04^b^	2.32±0.05^a^

## 4 Experimental Design, Materials and Methods

### 4.1 Collection of microalgae

Pure isolates of *Gonyostomum* sp. and *Tetraedron* sp. were collected from Microalgae Research Corner, Department of Aquaculture, Chattogram Veterinary and Animal Sciences University, Chattogram, Bangladesh. *Gonyostomum* sp. and *Tetraedron* sp. were marine (15 ppt) and freshwater microalgae mainly sourced from the Naf River estuary and Kaptai Lake respectively. Pure isolates were sub cultured in 50 mL, 100 mL conical flasks and finally scaled up to 500 mL and 1000 mL volume for the constant supply of pure stock. Liquid media such as Conway [5] and BBM [6] media were used to culture *Gonyostomum* sp. and *Tetraedron* sp. respectively. Stock cultures underwent routine microscopic observations to detect any signs of contamination.

### 4.2 Determination of growth curve

*Gonyostomum* sp. and *Tetraedron* sp. cultures were grown at a temperature of 24 ± 1 °C in a 350 mL culture volume of a sterile 500 mL borosilicate Erlenmeyer flask for each species with three replicates where 2% pure culture stocks were added. Microalgae cultures were maintained at 24 hr light condition at 150 µEm^−2^s^−1^ intensity with continuous gentle aeration at a rate of 4.53 ± 0.53 mg/L. The experiment was continued until the death phase and finally completed the growth curve depending on cell density (cells.ml^−1^) and optical density (absorbance).

#### 4.2.1 Determination of cell density

Microalgae cell count was carried out every day by using a Neubauer hemacytometer (0.0025 mm^2^, 0.1 mm deep chambers, Assistent, Germany). The microalgae cells were counted by using the formulae described by Lavens and Sorgeloos [7]:

#### 4.2.2 Determination of maximum absorbance (optical density)

Optical density (OD) was measured every day for the growth curve analysis. Maximum absorbance value for each microalga was used to perform the growth curve by OD. Maximum absorbance was measured at the wavelength of 300 nm for *Gonyostomum* sp. and 342 nm for *Tetraedron* sp. as these wavelengths gave maximum absorbance when the culture samples were scanned between 300 and 700 nm, using a spectrophotometer (NanoDrop Spectrophotometer, Model-Nanoplus, Germany).

### 4.3 Growth parameters

Specific growth rates (SGR) and Cell doublings per day were calculated according to Daniel and Srivastava [8] and cell duplication time was calculated according to Chiu et al. [9].

• Specific growth rates /SGR (r) = $\frac{\ln\text{Nt}-\ln\text{No}}{\Delta t}$

Where, Nt is the final cell count and No is the initial cell count; t is the number of days.

• Cell duplication time td= 0.693/µ, where µ=growth rate constant
• Cell doublings per day (K) = $\frac{\ln\text{Nn}-\ln\text{Ni}}{\text{ln}2(\text{tn}-\text{ti})}$

Here, Nn is the final cell count and Ni is the initial cell count; tn = final time in days and ti= initial time in days.

### 4.4 Determination of productivity

Volumetric productivity [10], areal productivity [11] and lipid productivity [12] were calculated according to the following equations. Productivity data was calculated at the end of the exponential phase.

• Volumetric productivity/VP (mg L^−1^ day^−1^) = $\frac{\left(X_{2}-X_{1}\right)}{\left(t_{2}-t_{1}\right)}$

Here, X_1_ and X_2_ were the biomass concentrations (mg L^−1^) on days t_1_ (start of study) and t_2_ (end of the study).

• AP (mg cm^−2^ day^−1^) = $\frac{\text{VP}\;\times \;V}{A}$

Where, VP = volumetric productivity, V = total volume of the culture, A = surface area occupied ground.

• LP (mg L^−1^ day^−1^) = $\frac{\text{Volumetric}\;\text{Productivity}\;\left(\text{mg}\;L^{-1}\;day^{-1}\right)\;x\;\left(\%\;\text{lipid}\right)}{100}$

Where, LP = Lipid productivity of the PBR and, % lipid = lipid content.

### 4.5 Determination of proximate compositions

#### 4.5.1 Protein determination

Protein was determined according to Lowry et al. [13]. 5 mg of dried biomass was dissolved in 25 mL distilled water and well mixed (tissue homogenizer). 0.5 mL solution was taken from that 25 mL solution and 0.5 mL of 1 N NaOH was added. The mixture was kept in a hot water bath (100 °C) for 5 min. Then, the samples were transferred to cold water bath. 10 min after cooling, 2.5 mL mixed reagent, mixed) was added to the sample. After mixing by a vortex mixture, 0.5 mL Folin reagent was added. Subsequently, the samples were kept in a dark place for 30 min and spectrophotometric analysis was performed at 750 nm wavelength.

#### 4.5.2 Carbohydrate determination

Carbohydrate was determined according to Dubois et al. [14]. 25 mL Microalgae solution was prepared following the same procedure as for the protein content. Then, 1 mL solution was taken and 1 mL of 5% phenol and 5 mL of sulfuric acid were added into it maintaining every 30 s reaction period. Then, the samples were placed in the cold-water bath. After cooling to the room temperature, spectrophotometric absorbances were measured at 488 nm wavelength.

#### 4.5.3 Lipid determination

Lipid was determined according to Bligh and Dyer [15]. Aluminum dishes were labeled and weighed as initial weight. 50 mg of each sample was taken in a centrifuge tube, and diluted into 5x volume using distilled water. Then, 3 mL methanol: chloroform (2:1, v/v) was added and mixed (tissue homogenizer) properly. After that, all the tubes were centrifuged (1000 rpm for 4 min at 4 °C). Then, the supernatants were transferred into clean tubes by clean Pasteur pipette. Again, in the test tubes, 3 mL of methanol: chloroform (2:1, v/v) was mixed (tissue homogenizer) properly and centrifuged at the same conditions, and the new supernatants were transferred to the previous supernatant containing tubes. 1.5 mL of 0.9% NaCl was poured into the combined supernatant tubes, and mixed well through vortex mixture and then placed in the refrigerator for 1 hour at 4 °C. Then the tubes were centrifuged (1000 rpm for 10 min at 4 °C), resulting in two separate layers. Methanol and chloroform containing upper layer was discarded and the lower layer was transferred to the previously prepared aluminum dish. Then, the solvent was evaporated using a hot air oven at 60 °C. After that, the aluminum dishes were weighed to obtain the final weight. Lipid content was calculated by subtracting the initial weight from the final weight.

### 4.6 Biochemical composition

#### 4.6.1 Determination of fatty acid

Fatty acids were analyzed by the “Two steps transesterification (2TE)” method after a little modification from Griffiths et al*.* [16]. By mixing 70 ml diethyl ether into 500 mg microalgae powder, lipid was extracted by using Digital Soxhlet Apparatus (FOOD ALYTRD40). After extraction, solvent was removed using Hot Air Oven at 60 °c. After that, 1.5 ml methanolic NaOH was poured into lipid extract and mixed in Sonicator at 80 °c for 5 min. After cooling to 25 °C, 2 ml BF_3_ methanol was added to the mixture and sonicated (80 °c for 30 min). After cooling to 25, 1 ml isooctane and 5 ml saturated NaCl was added and mixed through well shaking. The upper fatty-acid methyl-esters (FAMEs) organic layer was transferred to a new tube and 1 ml sample was taken into vial for fatty acid methyl-esters analysis by Gas Chromatography Mass Spectrophotometry (GC-2020plus, SHIMADZU, Japan). FAMEs were separated with a capillary column (length 30 m, internal diameter 0.25 mm, film thickness 0.15 µm, phase ratio 250). Helium gas was used at a flow rate of 1.42 ml/min as a carrier gas. The column temperature program was as follows: 180 °C to 280 °C at 5 °C /min and then held at 280 °C. FAMEs were detected by comparing retention time with standard (FAME mix C8-C24; Sigma- Aldrich; Germany).

#### 4.6.2 Determination of amino acid

The Moore and Stein technique [17] was slightly modified to identify amino acids. One gram dried microalgae was first hydrolyzed for 24 h at 110 °C in 25 mL of previously prepared acidic hydrolysis solution (6 M HCl + 0.1% phenol). The samples were stabilized using a little quantity of SDB/Na (Sample Dilution Buffer) after cooling. The pH of the samples was then adjusted using a basic neutralizing agent between 2.1 and 2.3 range. The hydrolysates were then filtered and diluted with SDB/Na before being placed in the injection vials. SYKAM S 433 amino acid analyzer with UV detector was used for the analysis. Nitrogen gas was employed as the carrier gas (0.5 mL/min flow rate, 3% reproducibility, at 60 °C). Sigma-Aldrich, Germany's AA-S-18 standard wease is used to measure the concentration of amino acids. Amino acids were measured in mg/g, which was then converted to the percent of all amino acids.

### 4.7 Pigment content

#### 4.7.1 Determination of chlorophyll

Chlorophyll concentration was determined according to Jenkins [18]. The clean extract was transferred to a 1 cm cuvette and OD was measured at 750, 664, 647 and 630 nm wavelength. OD at 664, 647, and 630 nm were used for chlorophyll determination where, OD at 750 nm was used as turbidity correction factor and subtracted from each of the pigments OD values before using them in the equations. Concentrations of chlorophyll a and b in the extract were calculated by inserting the corrected optical densities in equations provided by Jeffrey and Humphrey [19]:

#### 4.7.2 Determination of carotenoids

1 mL aliquot of the algal suspension from each culture was taken at their stationary phase and centrifuged at 1000 g for 5 min. Then, the obtained pellet was extracted with 3 mL of ethanol: hexane 2:1 (v/v). After that, 2 mL of water and 4 mL hexane (Sigma, USA) were added to the mixture, shaken vigorously and centrifuged again at 1000 × *g* for 5 min. Finally, absorbance of the separated hexane layer was determined using spectrophotometer at a wavelength of 450 nm. The amount of extracted carotene from the samples in micrograms was determined by multiplying the absorbance (A_450)_ with 25.2 (Shaish, 1992) [20].

#### 4.7.3 Determination of phycobiliproteins

As phycobiliproteins estimation required dried biomass, the cultures were harvested by centrifugation at 6000 rpm for 5 min and the harvested pellets were dried at 40 °C for overnight. 40 mg of dried powder was then soaked in 10 mL phosphate buffer (pH 7.0; 0.1 M), mixed properly using vortex mixture, and then stored for 24 hr at 4 °C. After that, the samples were centrifuged at 6000 rpm for 10 min. Finally, the supernatant was collected and spectrophotometric (Nano Drop Spectrophotometer, Model-Nanoplus, Germany) absorbance was measured against the phosphate buffer solution as blank at the wavelength of 562, 615, 652 and 720 nm where 720 nm measured the absorbance of the cellular debris.

The amount of phycocyanin (PC), and allophycocyanin (APC), phycoerythrin (PE) in the sample was calculated from the absorbance according to Siegelman and Kycia [21].

Total phycocyanin, phycoerythrin, and allophycocyanin (mg/g) were calculated according to Silveira et al. [22].

### 4.8 Statistical analysis

All statistical analyses regarding the growth and productivity parameters; proximate composition, biochemical composition and pigment contents were performed using the IBM SPSS (v. 26.0). The collected data were subjected to *t*-test at *p* ˂ 0.05 for all analyses.

## Limitations

Not applicable.

## Ethical Statement

No conflicts, informed consent, or human or animal rights are applicable to this study.

## CRediT authorship contribution statement

**Proma Dey:** Methodology, Data curation, Writing – original draft. **Jannatul Nayeem:** Data curation, Formal analysis. **Sumit Kanti Dey:** Data curation. **Dipa Debi:** Formal analysis. **Nusrat Zaman Zemi:** Formal analysis. **S. M. Rashadul Islam:** Data curation. **Rezaul Karim:** Writing – review & editing. **Helena Khatoon:** Conceptualization, Funding acquisition, Supervision, Resources, Validation, Writing – review & editing.

## Data Availability

Comparative dataset on productivity, proximate, biochemical and pigment content of marine Gonyostomum sp. and freshwater Tetraedron sp. microalgae (Original data) (Mendeley Data)
